# Supplementary material for: Estimating Dengue Transmission Intensity in China Using Catalytic Models Based on Serological Data
Source: Trop Med Infect Dis. 2023 Feb 14;8(2):116. doi: 10.3390/tropicalmed8020116 (PMC9967418; doi:10.3390/tropicalmed8020116)
Supplement: Supplementary file 1 [file tropicalmed-08-00116-s001.zip › SupplementaryS2.pdf]

Supplementary Material

Estimating Dengue transmission intensity in China using dynamics catalytic models based on serological data

Ning Li [1,2], Haidong Li [1,3], Zhengji Chen [1,4], Huan Xiong [1], Zhibo Li [1], Tao Wei [5], Wei Liu [1], Xu-Sheng Zhang [6,*]

[1] School of Public Health, Kunming Medical University, Kunming, Yunnan, P. R. China
[2] Centers for Disease Control and Prevention of Yunnan province, Kunming, P. R. China
[3] The Affiliated Hospital of Stomatology, Chongqing Medical University, Chongqing 401147, P.R. China
[4] Kunming Centers for Disease Control and Prevention, Kunming, Yunnan province, P. R. China
[5] Library, Kunming Medical University, Kunming, Yunnan, P. R. China
[6] Statistics, Modelling and Economics Department, Data, Analytics & Surveillance, UK Health Security Agency, London, UK.

[*]**Corresponding author:** Xu-Sheng.Zhang@ukhsa.gov.uk (XZ)

S2 Figures and Tables

Content

	page
**1.Figures**	**2**
1.1 Figure S1 The fitting curves of Model A to 31 datasets	3
1.2 Figure S2 The fitting curves of Model B to 31 datasets	4
1.3 Figure S3 The fitting curves of Model C to 31 datasets	5
1.4 Figure S4 The fitting curves of Model D to 31 datasets	6
1.5 Figure S5 Distribution of R_0 in different study years	7
**2.Tables**	**8**
2.1 Table S1 Summary of model parameter estimates for Model A	8
2.2 Table S2 Summary of model parameter estimates for Model B	10
2.3 Table S3 Summary of model parameter estimates for Model C	12
2.4 Table S4 Summary of model parameter estimates for Model D	14
2.5 Table S5 Comparison of DIC among four models	16
**3. Reference**	**18**

1. Figures

Note that in Figures S1-S4, the blue dotted line, the green bold solid line, and the red dotted line are the 2.5%, 50% and 97.5% of the model fitting curves, respectively, and the red dots are the observation data. To make the graphs neat, the following abbreviations are used:

GZ Huangpu [4] represents Huangpu District in Guangzhou city;

QZ/FCG/HP [9] stands for Qinzhou City, Fangchenggang City and Hepu County in Guangxi;

GY/CJ/LD [15] represents Yunyan District of Guiyang City, Congjiang County of Southeast Guizhou Province and Luodian County of Guizhou province;

TP/TY/TN [20] indicates Taipei, Taoyuan and Tainan of Taiwan Province.

Figure S1. The fitting curves from the constant force of infection model (Model A) to 31 datasets

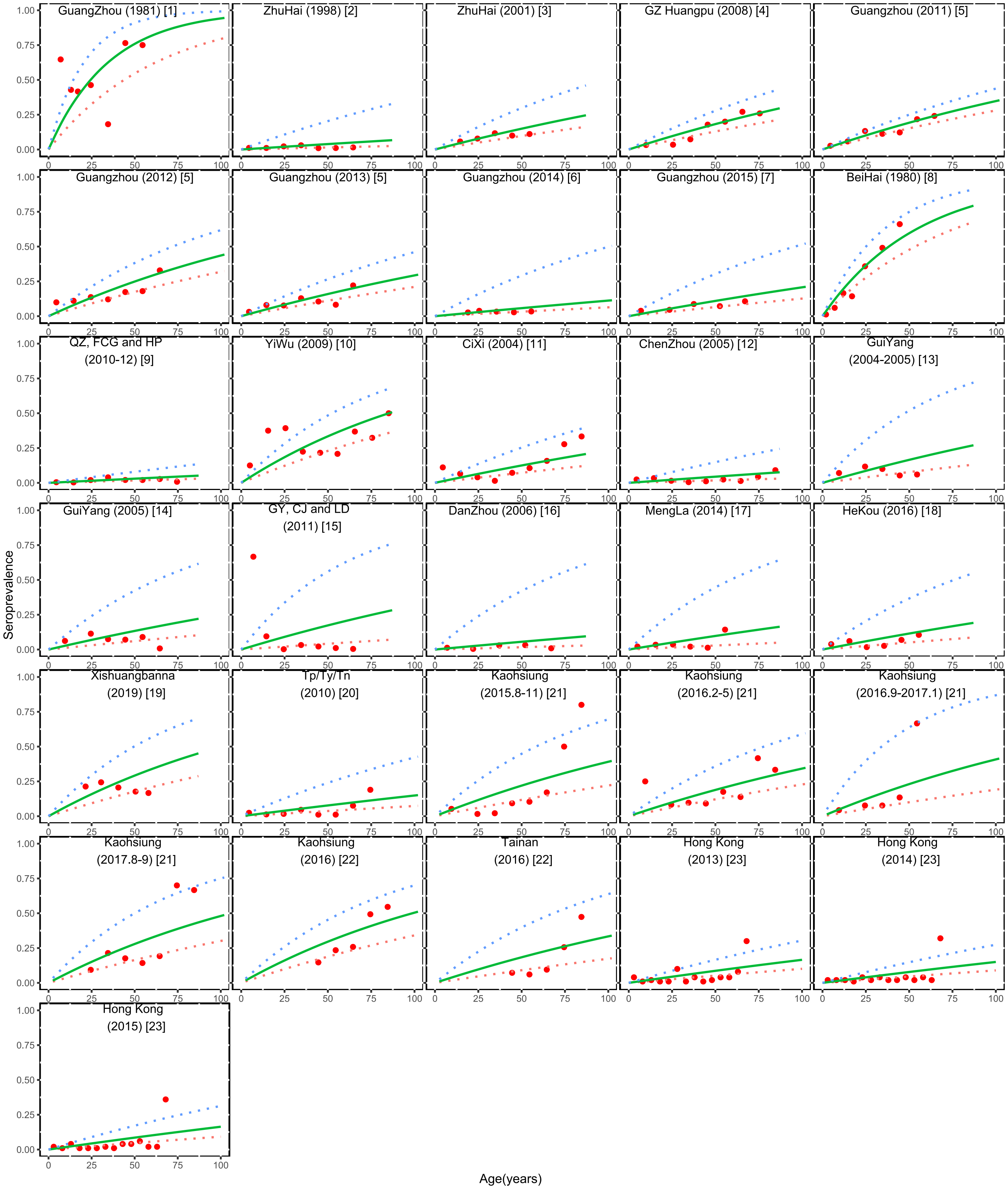

Figure S2. The fitting curves from the antibody protection decay model (Model B) to 31 datasets

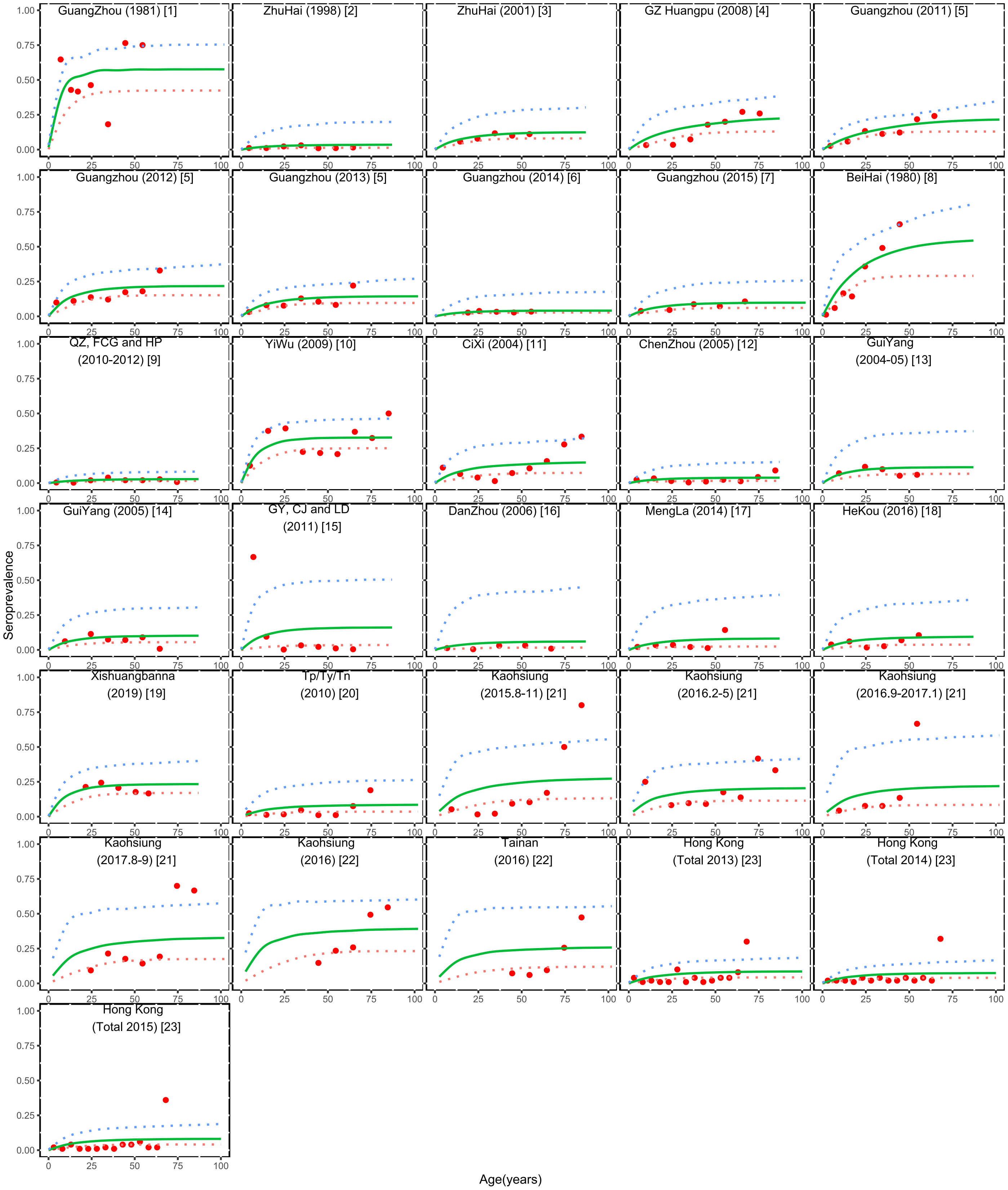

Figure S3. The fitting curves from the constant force of infection and age threshold model (Model C) to 31 datasets

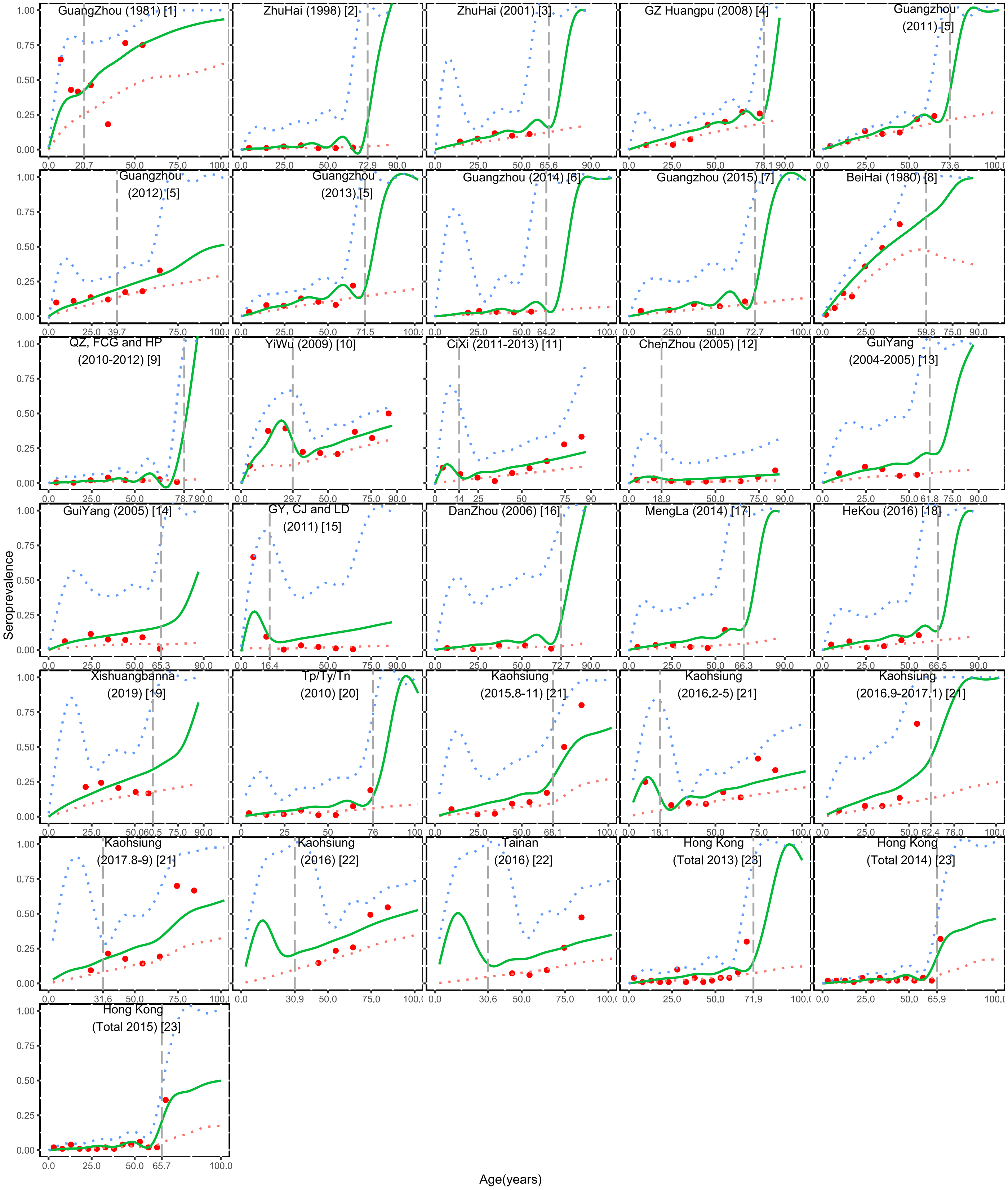

Figure S4. The fitting curves from the antibody protection decay and age threshold model (Model D) to 31 datasets

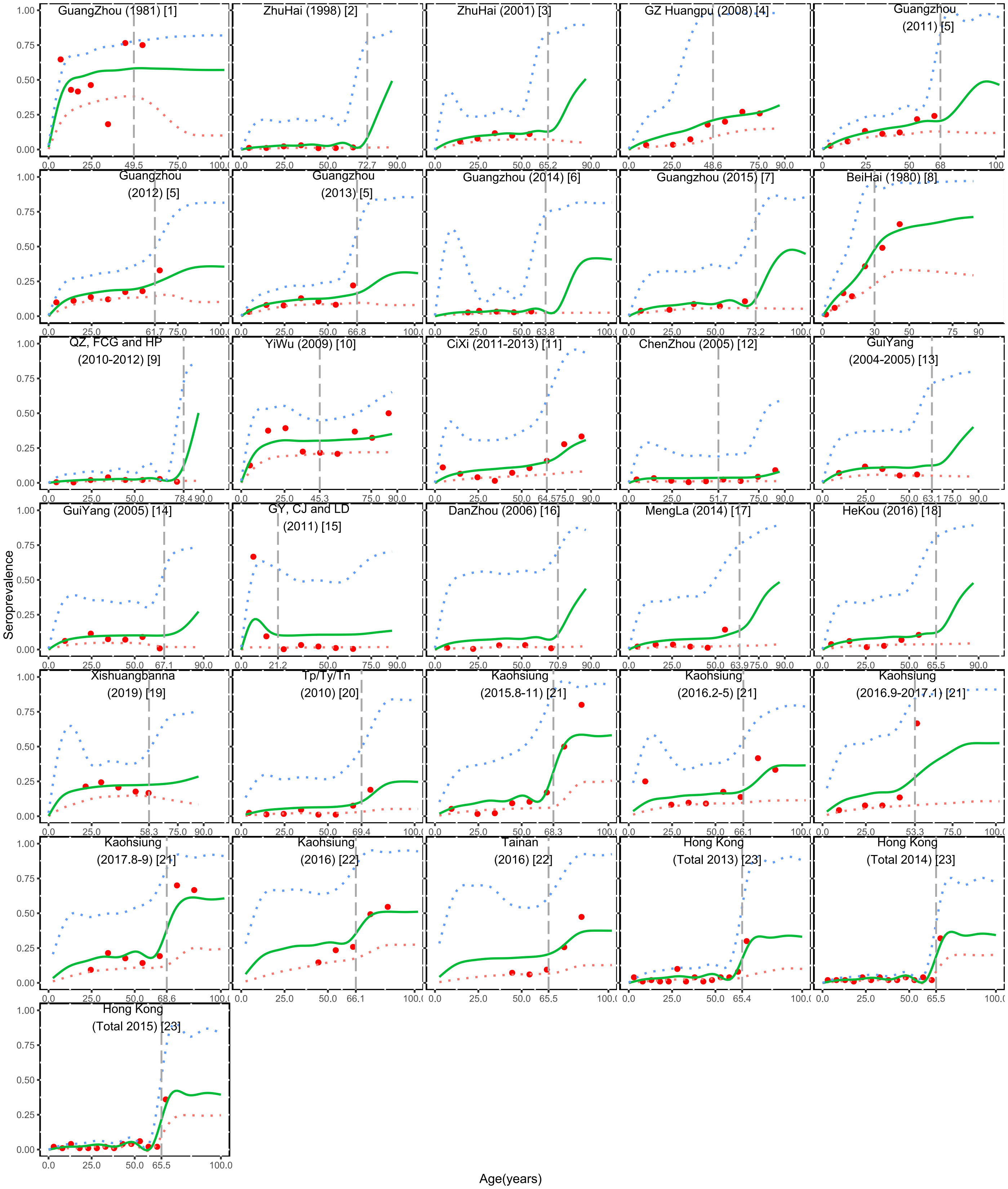

Figure S5. Distribution of R_0 in different study years

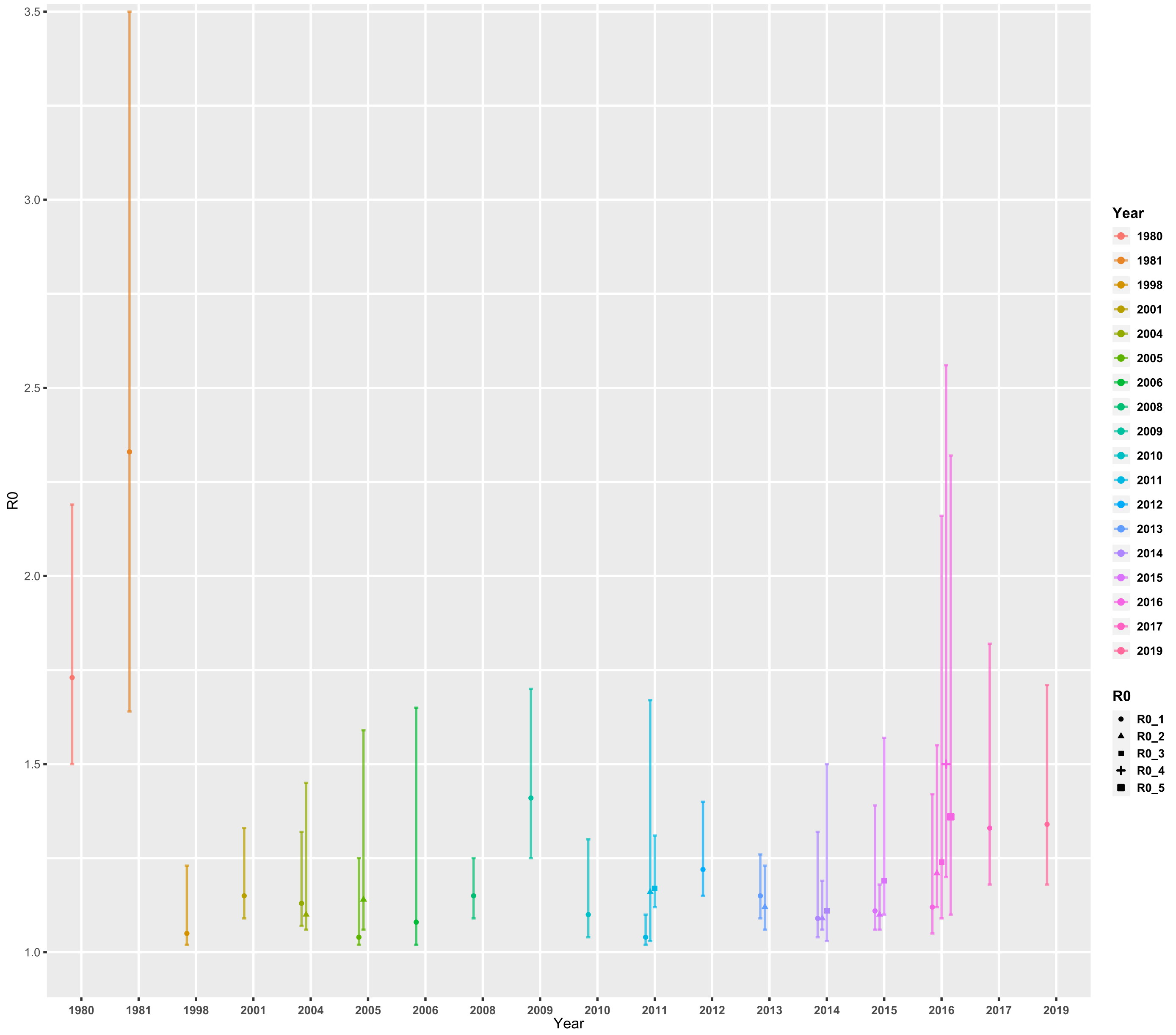

Note: R_{0_1}, R_{0_2}, R_{0_3}, R_{0_4}, R_{0_5} are the basic reproduction numbers of different data sets in the same study year, respectively.

2. Tables

Table S1: Summary of model parameter estimates for the constant force of infection model (Model A)

Survey region	Reference	Survey year	FOI (λ , 95%CrI)	R_0 (95%CrI)	γ (95%CrI)	-LnL	DIC
**Guangdong Province**							
**Guangzhou**	Huang Y et al. [1]	1981	0.0285 (0.0158 , 0.0481)	2.35 (1.65 , 3.75)	0.20 (0.05 , 0.45)	123.54	249.46
**Zhuhai**	Li Z et al. [2]	1998	0.0008 (0.0003 , 0.0046)	1.03 (1.01 , 1.16)	0.04 (0.00 , 0.29)	36.20	71.95
**Zhuhai**	Yang Z et al. [3]	2001	0.0032 (0.0020 , 0.0071)	1.11 (1.07 , 1.25)	0.03 (0.00 , 0.20)	172.46	345.07
**# GZ Huangpu**	Zheng X et al. [4]	2008	0.0040 (0.0028 , 0.0065)	1.14 (1.09 , 1.23)	0.03 (0.00 , 0.14)	140.82	282.69
**Guangzhou**	Cao Y et al. [5]	2011-13					
		2011	0.0043 (0.0033 , 0.0058)	1.15 (1.12 , 1.21)	0.01 (0.00 , 0.05)	603.41	1208.26
		2012	0.0057 (0.0038 , 0.0096)	1.21 (1.14 , 1.37)	0.05 (0.01 , 0.18)	519.23	1040.36
		2013	0.0035 (0.0024 , 0.0062)	1.12 (1.08 , 1.23)	0.03 (0.00 , 0.12)	392.85	787.48
**Guangzhou**	Li S et al. [6]	2014	0.0012 (0.0007 , 0.0069)	1.04 (1.02 , 1.26)	0.05 (0.00 , 0.36)	582.55	1164.52
**Guangzhou**	Jing Q et al. [7]	2015	0.0023 (0.0014 , 0.0072)	1.08 (1.05 , 1.27)	0.05 (0.00 , 0.28)	207.67	415.95
**Guangxi Province**							
**Beihai**	Tian X et al. [8]	1980	0.0181 (0.0130 , 0.0277)	1.73 (1.50 , 2.19)	0.06 (0.00 , 0.20)	208.16	418.28
**$ QZ/FCG/HP**	Zhou K et al. [9]	2010-12	0.0006 (0.0004 , 0.0017)	1.02 (1.01 , 1.05)	0.02 (0.00 , 0.11)	185.91	372.55
**Zhejiang Province**							
**Yiwu**	Sun J et al. [10]	2009	0.0081 (0.0052 , 0.0133)	1.30 (1.19 , 1.52)	0.10 (0.02 , 0.25)	221.27	444.86
**Cixi**	Cen D et al. [11]	2004	0.0027 (0.0015 , 0.0058)	1.11 (1.06 , 1.24)	0.05 (0.00 , 0.20)	127.22	256.27
**Hunan Province**							
**Chenzhou**	Gao L et.al. [12]	2005	0.0009 (0.0004, 0.0032)	1.03 (1.01 , 1.11)	0.05 (0.00 , 0.23)	51.99	105.27
**Guizhou Province**							
**Guiyang**	Gao R et al. [13]	2004-05	0.0036 (0.0016 , 0.0146)	1.13 (1.06 , 1.59)	0.10 (0.02 , 0.50)	672.49	1346.25
**Guiyang**	Tian H et al. [14]	2005	0.0029 (0.0012 , 0.0110)	1.10 (1.04 , 1.43)	0.10 (0.01 , 0.50)	204.78	410.90
**& GY/CJ/LD**	Jiang W et al. [15]	2011	0.0038 (0.0008 , 0.0164)	1.14 (1.03 , 1.67)	0.30 (0.05 , 0.70)	59.94	120.93
**Hainan Province**							
**Danzhou**	Jin Y et al. [16]	2006	0.0011 (0.0003 , 0.0109)	1.04 (1.01 , 1.38)	0.12 (0.00 , 0.60)	45.15	89.21
**Yunnan Province**							
**Mengla**	Lu Y et al. [17]	2014	0.0021 (0.0006 , 0.0118)	1.07 (1.02 , 1.43)	0.10 (0.00 , 0.46)	26.48	52.97
**Hekou**	Pu L et al. [18]	2016	0.0024 (0.0010 , 0.0091)	1.08 (1.03 , 1.33)	0.07 (0.00 , 0.36)	39.51	79.88

**Xishuangbanna**	Li L et al. [19]	2019	0.0069 (0.0039 , 0.0140)	1.28 (1.15 , 1.61)	0.10 (0.02 , 0.36)	1178.3	2358.14
**Taiwan Province**							
**% TP/TY/TN**	Lee YH et al. [20]	2010	0.0016 (0.0008 , 0.0055)	1.07 (1.03 , 1.25)	0.10 (0.01 , 0.30)	184.93	374.11
**Kaohsiung**	Tsai JJ et al. [21]	2015-17					
		2015.8-11	0.0050 (0.0025 , 0.0119)	1.23 (1.11 , 1.60)	0.20 (0.04, 0.44)	128.97	260.08
		2016.2-5	0.0042 (0.0026 , 0.0089)	1.19 (1.11 , 1.43)	0.06 (0.00, 0.26)	111.18	223.49
		2016.9-2017.1	0.0052 (0.0021 , 0.0203)	1.24 (1.09 , 2.16)	0.10 (0.00, 0.52)	78.47	157.37
		2017.8-9	0.0066 (0.0036 , 0.0139)	1.31 (1.16 , 1.72)	0.10 (0.01, 0.39)	69.62	141.19
**Kaohsiung and Tainan**	Pan YH et al. [22]	2016					
**Kaohsiung**			0.0070 (0.0042 , 0.0121)	1.33 (1.19 , 1.61)	0.10 (0.02, 0.36)	961.22	1924.29
**Tainan**			0.0041 (0.0019 , 0.0102)	1.18 (1.08 , 1.50)	0.20 (0.04, 0.48)	858.61	1719.13
**Hong Kong**	Lee P et al. [23]	2013-15					
		2013	0.0018 (0.0011 , 0.0036)	1.08 (1.05 , 1.16)	0.06 (0.01 , 0.16)	132.91	267.96
		2014	0.0016 (0.0009 , 0.0032)	1.07 (1.04 , 1.14)	0.05 (0.01 , 0.14)	121.16	244.59
		2015	0.0018 (0.0010 , 0.0038)	1.08 (1.04 , 1.17)	0.10 (0.02 , 0.20)	118.30	238.80

Note: # GZ Huangpu represents Huangpu District in Guangzhou city; $ QZ/FCG/HP stands for Qinzhou City, Fangchenggang City and Hepu County in Guangxi; & GY/CJ/LD represents Yunyan District of Guiyang City, Congjiang County of Southeast Guizhou Province and Luodian County of Guizhou Province; % TP/TY/TN indicates Taipei, Taoyuan and Tainan of Taiwan Province.

Table S2: Summary of model parameter estimates of the antibody protection decay model (Model B)

Survey region	Reference	Survey year	α (95%CrI)	FOI (λ, 95%CrI)	R_0 (95%CrI)	γ (95%CrI)	-LnL	DIC
**Guangdong Province**								
**Guangzhou**	Huang Y et al. [1]	1981	0.07 (0.02 , 0.10)	0.0910 (0.0368 , 0.1456)	2.16 (1.64 , 3.05)	0.10 (0.03 , 0.41)	121.66	245.36
**Zhuhai**	Li Z et al. [2]	1998	0.07 (0.01 , 0.10)	0.0024 (0.0007 , 0.0162)	1.03 (1.01 , 1.18)	0.05 (0.00 , 0.37)	35.14	69.96
**Zhuhai**	Yang Z et al. [3]	2001	0.06 (0.01 , 0.10)	0.0083 (0.0034 , 0.0233)	1.11 (1.07 , 1.31)	0.03 (0.00 , 0.22)	171.83	343.78
**# GZ Huangpu**	Zheng X et al. [4]	2008	0.03 (0.00 , 0.10)	0.0094 (0.0037 , 0.0326)	1.17 (1.10 , 1.40)	0.07 (0.00 , 0.27)	143.90	287.56
**Guangzhou**	Cao Y et al. [5]	2011-13						
		2011	0.03 (0.00 , 0.09)	0.0077 (0.0042 , 0.0223)	1.16 (1.12 , 1.27)	0.03 (0.00 , 0.12)	605.33	1210.97
		2012	0.07 (0.01 , 0.10)	0.0176 (0.0068 , 0.0367)	1.22 (1.15 , 1.41)	0.05 (0.01 , 0.19)	518.33	1038.56
		2013	0.06 (0.01 , 0.10)	0.0100 (0.0041 , 0.0229)	1.13 (1.09 , 1.25)	0.03 (0.00 , 0.14)	391.98	785.54
**Guangzhou**	Li S et al. [6]	2014	0.07 (0.01 , 0.10)	0.0030 (0.0014 , 0.0130)	1.03 (1.02 , 1.16)	0.02 (0.00 , 0.21)	579.54	1156.73
**Guangzhou**	Jing Q et al. [7]	2015	0.07 (0.01 , 0.10)	0.0069 (0.0027 , 0.0196)	1.09 (1.05 , 1.25)	0.03 (0.00 , 0.23)	205.89	411.70
**Guangxi Province**								
**Beihai**	Tian X et al. [8]	1980	0.02 (0.00 , 0.10)	0.0269 (0.0148 , 0.0790)	1.62 (1.32 , 2.18)	0.10 (0.01 , 0.37)	211.03	422.83
**$ QZ/FCG/HP**	Zhou K et al. [9]	2010-12	0.07 (0.02 , 0.10)	0.0019 (0.0008 , 0.0062)	1.02 (1.01 , 1.06)	0.02 (0.00 , 0.10)	184.17	368.74
**Zhejiang Province**								
**Yiwu**	Sun J et al. [10]	2009	0.08 (0.03 , 0.10)	0.0366 (0.0168 , 0.0636)	1.41 (1.28 , 1.69)	0.05 (0.00 , 0.17)	217.26	436.63
**Cixi**	Cen D et al. [11]	2004	0.06 (0.00 , 0.10)	0.0094 (0.0025 , 0.0300)	1.13 (1.06 , 1.36)	0.10 (0.01 , 0.32)	128.71	258.88
**Hunan Province**								
**Chenzhou**	Gao L et.al. [12]	2005	0.08 (0.02 , 0.10)	0.0028 (0.0009 , 0.0113)	1.03 (1.01 , 1.13)	0.04 (0.00 , 0.23)	50.45	101.58
**Guizhou Province**								
**Guiyang**	Gao R et al. [13]	2004-05	0.08 (0.02 , 0.10)	0.0092 (0.0038 , 0.0358)	1.10 (1.06 , 1.45)	0.08 (0.01 , 0.38)	669.62	1339.97
**Guiyang**	Tian H et al. [14]	2005	0.08 (0.02 , 0.10)	0.0082 (0.0032 , 0.0316)	1.09 (1.05 , 1.34)	0.08 (0.00 , 0.39)	201.53	403.67
**& GY/CJ/LD**	Jiang W et al. [15]	2011	0.08 (0.02 , 0.10)	0.0128 (0.0021 , 0.0697)	1.15 (1.03 , 1.81)	0.30 (0.04 , 0.71)	58.17	117.36
**Hainan Province**								
**Danzhou**	Jin Y et al. [16]	2006	0.07 (0.01 , 0.10)	0.0039 (0.0008 , 0.0455)	1.05 (1.01 , 1.55)	0.10 (0.00 , 0.64)	45.10	88.84
**Yunnan Province**								
**Mengla**	Lu Y et al. [17]	2014	0.07 (0.01 , 0.10)	0.0052 (0.0013 , 0.0389)	1.06 (1.02 , 1.42)	0.10 (0.00 , 0.50)	26.05	51.99
**Hekou**	Pu L et al. [18]	2016	0.07 (0.01 , 0.10)	0.0065 (0.0018 , 0.0363)	1.08 (1.03 , 1.38)	0.08 (0.00 , 0.43)	39.24	79.23
**Xishuangbanna**	Li L et al. [19]	2019	0.08 (0.02 , 0.10)	0.0229 (0.0097 , 0.0450)	1.25 (1.17 , 1.49)	0.04 (0.03 , 0.23)	1174.73	2349.94
**Taiwan Province**								

**% TP/TY/TN**	Lee YH et al. [20]	2010	0.07 (0.01 , 0.10)	0.0056 (0.0016 , 0.0228)	1.07 (1.03 , 1.27)	0.10 (0.02 , 0.38)	185.17	371.79
**Kaohsiung**	Tsai JJ et al. [21]	2015-17						
		2015.8-11	0.05 (0.00 , 0.10)	0.0167 (0.0042 , 0.0682)	1.28 (1.12 , 1.87)	0.20 (0.07 , 0.54)	130.86	263.05
		2016.2-5	0.07 (0.01 , 0.10)	0.0151 (0.0048 , 0.0487)	1.21 (1.11 , 1.56)	0.10 (0.00 , 0.31)	111.74	225.02
		2016.9-2017.1	0.06 (0.07 , 0.10)	0.0143 (0.0039 , 0.0785)	1.22 (1.08 , 2.03)	0.10 (0.00 , 0.55)	78.95	158.18
		2017.8-9	0.06 (0.00 , 0.10)	0.0239 (0.0059 , 0.0851)	1.37 (1.17 , 2.04)	0.20 (0.02 , 0.46)	71.24	143.92
**Kaohsiung and Tainan**	Pan YH et al. [22]	2016						
**Kaohsiung**			0.06 (0.01 , 0.10)	0.0366 (0.0079 , 0.1062)	1.51 (1.24 , 2.28)	0.20 (0.04 , 0.46)	963.13	1927.82
**Tainan**			0.07 (0.01 , 0.10)	0.0204 (0.0043 , 0.0883)	1.28 (1.11 , 2.07)	0.20 (0.06 , 0.59)	859.77	1721.15
**Hong Kong**	Lee P et al. [23]	2013-15						
		2013	0.06 (0.01 , 0.10)	0.0053 (0.0018 , 0.0148)	1.07 (1.04 , 1.17)	0.10 (0.02 , 0.21)	133.24	268.73
		2014	0.07 (0.01 , 0.10)	0.0051 (0.0018 , 0.0123)	1.07 (1.04 , 1.14)	0.10 (0.02 , 0.17)	120.86	244.05
		2015	0.07 (0.01 , 0.10)	0.0054 (0.0019 , 0.0146)	1.07 (1.04 , 1.17)	0.10 (0.02 , 0.26)	118.23	238.72

Note: # GZ Huangpu represents Huangpu District in Guangzhou city; $ QZ/FCG/HP stands for Qinzhou City, Fangchenggang City and Hepu County in Guangxi; & GY/CJ/LD represents Yunyan District of Guiyang City, Congjiang County of Southeast Guizhou Province and Luodian County of Guizhou Province; % TP/TY/TN indicates Taipei, Taoyuan and Tainan of Taiwan Province.

Table S3: Summary of model parameter estimates from constant force of infection and age threshold model (Model C)

Survey region	Reference	Survey year	A_{crit} (95%CI)	λ_1 (95%CrI)	λ_2 (95%CrI)	R_0 (95%CrI)	γ (95%CrI)	-LnL	DIC
**Guangdong Province**									
**Guangzhou**	Huang Y et al. [1]	1981	20.7 (9.9 , 82.1)	0.0538 (0.0184 , 0.1440)	0.0269 (0.0094 , 0.1348)	2.33 (1.64 , 3.50)	0.10 (0.02 , 0.41)	122.80	240.45
**Zhuhai**	Li Z et al. [2]	1998	72.9 (11.6 , 83.8)	0.0009 (0.0003 , 0.0236)	0.0595 (0.0004 , 0.1447)	1.05 (1.02 , 1.23)	0.05 (0.00 , 0.39)	35.26	61.68
**Zhuhai**	Yang Z et al. [3]	2001	65.6 (10.3 , 83.3)	0.0034 (0.0021 , 0.1238)	0.0585 (0.0022 , 0.1448)	1.15 (1.09 , 1.33)	0.04 (0.00 , 0.23)	172.53	294.39
**# GZ Huangpu**	Zheng X et al. [4]	2008	78.1 (9.9 , 84.1)	0.0041 (0.0019 , 0.0533)	0.0428 (0.0028 , 0.1446)	1.15 (1.09 , 1.25)	0.03 (0.00 , 0.16)	140.92	177.13
**Guangzhou**	Cao Y et al. [5]	2011-13							
		2011	73.6 (15.6 , 84.0)	0.0043 (0.0034 , 0.0073)	0.0669 (0.0032 , 0.1465)	1.18 (1.13 , 1.25)	0.01 (0.00 , 0.06)	603.45	1207.90
		2012	39.7 (9.9 , 83.1)	0.0078 (0.0041 , 0.0598)	0.0071 (0.0034 , 0.1400)	1.22 (1.15 , 1.40)	0.05 (0.00 , 0.18)	518.73	955.25
		2013	71.5 (12.2 , 83.9)	0.0037 (0.0024 , 0.0175)	0.0532 (0.0022 , 0.1447)	1.15 (1.09 , 1.26)	0.03 (0.00 , 0.14)	392.87	717.46
**Guangzhou**	Li S et al. [6]	2014	64.2 (10.4 , 83.7)	0.0014 (0.0007 , 0.1331)	0.0476 (0.0007 , 0.1450)	1.09 (1.04 , 1.32)	0.05 (0.00 , 0.38)	582.51	966.85
**Guangzhou**	Jing Q et al. [7]	2015	72.7 (11.7 , 84.0)	0.0027 (0.0014 , 0.0308)	0.0517 (0.0014 , 0.1444)	1.11 (1.06 , 1.39)	0.06 (0.00 , 0.37)	207.78	355.98
**Guangxi Province**									
**Beihai**	Tian X et al. [8]	1980	59.8 (17.1 , 83.1)	0.0176 (0.0102 , 0.0277)	0.0560 (0.0054 , 0.1452)	1.80 (1.51 , 2.26)	0.05 (0.00 , 0.20)	208.12	417.75
**$ QZ/FCG/HP**	Zhou K et al. [9]	2010-12	78.7 (13.1 , 84.2)	0.0006 (0.0003 , 0.0064)	0.0606 (0.0003 , 0.1450)	1.04 (1.02 , 1.10)	0.02 (0.00 , 0.16)	185.87	193.52
**Zhejiang Province**									
**Yiwu**	Sun J et al. [10]	2009	29.7 (16.1 , 42.0)	0.0252 (0.0122 , 0.0559)	0.0061 (0.0043 , 0.0093)	1.41 (1.25 , 1.70)	0.04 (0.00 , 0.15)	215.09	433.33
**Cixi**	Cen D et al. [11]	2004	14.0 (9.7 , 81.9)	0.0238 (0.0016 , 0.1317)	0.0029 (0.0015 , 0.0205)	1.13 (1.07 , 1.32)	0.07 (0.00 , 0.24)	127.08	218.66
**Hunan Province**									
**Chenzhou**	Gao L et.al. [12]	2005	18.9 (9.9 , 75.4)	0.0055 (0.0009 , 0.0514)	0.0007 (0.0002 , 0.0044)	1.04 (1.01 , 1.20)	0.06 (0.00 , 0.32)	50.64	101.25
**Guizhou Province**									
**Guiyang**	Gao R et al. [13]	2004-05	61.8 (11.5 , 83.3)	0.0046 (0.0017 , 0.0489)	0.0393 (0.0012 , 0.1453)	1.18 (1.07 , 1.73)	0.10 (0.01 , 0.51)	672.33	1339.04
**Guiyang**	Tian H et al. [14]	2005	65.3 (11.4 , 83.6)	0.0047 (0.0014 , 0.0669)	0.0094 (0.0006 , 0.1426)	1.14 (1.06 , 1.59)	0.10 (0.01 , 0.55)	204.61	362.19
**& GY/CJ/LD**	Jiang W et al. [15]	2011	16.4 (9.9 , 80.8)	0.0469 (0.0020 , 0.1449)	0.0025 (0.0004 , 0.1269)	1.16 (1.03 , 1.67)	0.20 (0.02 , 0.65)	57.27	80.41
**Hainan Province**									
**Danzhou**	Jin Y et al. [16]	2006	72.7 (11.3 , 84.0)	0.0017 (0.0004 , 0.0706)	0.0462 (0.0006 , 0.1447)	1.08 (1.02 , 1.65)	0.10 (0.00 , 0.68)	46.05	53.5
**Yunnan Province**									
**Mengla**	Lu Y et al. [17]	2014	66.3 (11.1 , 83.4)	0.0024 (0.0007 , 0.0554)	0.0538 (0.0010 , 0.1448)	1.11 (1.03 , 1.50)	0.10 (0.00 , 0.50)	26.54	46.74
**Hekou**	Pu L et al. [18]	2016	66.5 (11.0 , 83.7)	0.0028 (0.0011 , 0.0409)	0.0551 (0.0012 , 0.1441)	1.12 (1.05 , 1.42)	0.08 (0.00 , 0.39)	39.58	72.62
**Xishuangbanna**	Li L et al. [19]	2019	60.5 (10.9 , 83.3)	0.0086 (0.0040 , 0.1340)	0.0199 (0.0031 , 0.1439)	1.34 (1.18 , 1.71)	0.10 (0.01 , 0.38)	1178.12	2321.00
**Taiwan Province**									

**% TP/TY/TN**	Lee YH et al. [20]	2010	76.00 (10.2,84.0)	0.0021 (0.0008 , 0.0545)	0.0262 (0.0009 , 0.1436)	1.10 (1.04 , 1.30)	0.10 (0.01 , 0.33)	184.95	278.49
**Kaohsiung**	Tsai JJ et al. [21]	2015-17							
		2015.8-11	68.1 (11.4 , 83.1)	0.0034 (0.0014 , 0.0782)	0.0099 (0.0031 , 0.0419)	1.19 (1.10 , 1.57)	0.10 (0.00 , 0.43)	127.49	238.28
		2016.2-5	18.1 (10.0 , 76.2)	0.0389 (0.0033 , 0.1195)	0.0039 (0.0023 , 0.0108)	1.23 (1.13 , 1.47)	0.05 (0.00 , 0.23)	109.39	220.42
		2016.9-2017.1	62.4 (12.8 , 83.3)	0.0057 (0.0021 , 0.0539)	0.0493 (0.0028 , 0.1449)	1.44 (1.14 , 2.38)	0.10 (0.00 , 0.51)	78.63	155.99
		2017.8-9	31.6 (10.3 , 81.5)	0.0097 (0.0026 , 0.1428)	0.0089 (0.0039 , 0.0370)	1.33 (1.18 , 1.82)	0.10 (0.00 , 0.38)	69.28	114.74
**Kaohsiung/Tainan**	Pan YH et al. [22]	2016							
**Kaohsiung**			30.9 (10.5 , 74.3)	0.0553 (0.0026 , 0.1447)	0.0073 (0.0043 , 0.0133)	1.50 (1.20 , 2.56)	0.10 (0.00 , 0.36)	961.12	1922.57
**Tainan**			30.6 (10.2 , 79.7)	0.0617 (0.0015 , 0.1461)	0.0042 (0.0019 , 0.0133)	1.36 (1.10 , 2.32)	0.10 (0.03 , 0.48)	858.53	1718.03
**Hong Kong**	Lee P et al. [23]	2013-15							
		2013	71.9 (44.8 , 83.8)	0.0017 (0.0008 , 0.0128)	0.0299 (0.0013 , 0.1450)	1.12 (1.06 , 1.23)	0.05 (0.00 , 0.18)	132.62	233.79
		2014	65.9 (30.2 , 81.5)	0.0010 (0.0005 , 0.0037)	0.0063 (0.0019 , 0.1252)	1.09 (1.06 , 1.19)	0.02 (0.00 , 0.09)	113.46	213.28
		2015	65.7 (17.6 , 81.8)	0.0009 (0.0005 , 0.0057)	0.0070 (0.0019 , 0.1263)	1.10 (1.06 , 1.18)	0.02 (0.00 , 0.12)	109.87	213.16

Note: # GZ Huangpu represents Huangpu District in Guangzhou city; $ QZ/FCG/HP stands for Qinzhou City, Fangchenggang City and Hepu County in Guangxi; & GY/CJ/LD represents Yunyan District of Guiyang City, Congjiang County of Southeast Guizhou Province and Luodian County of Guizhou Province; % TP/TY/TN indicates Taipei, Taoyuan and Tainan of Taiwan Province.

Table S4: Summary of model parameter estimates from antibody protection decay and age threshold model (Model D)

Survey region	Reference	Survey	A_{crit} (95%CrI)	α (95%CrI)	λ_1 (95%CrI)	λ_2 (95%CrI)	R_0 (95%CrI)	γ (95%CrI)	-LnL	DIC
**Guangdong Province**										
**Guangzhou**	Huang Y et al. [1]	1981	49.5<br>(10.9 , 82.8)	0.07<br>(0.02 , 0.10)	0.0904<br>(0.0361 , 0.1444)	0.0873<br>(0.0071 , 0.1471)	2.13<br>(1.58 , 3.06)	0.10<br>(0.02 , 0.40)	121.71	245.20
**Zhuhai**	Li Z et al. [2]	1998	72.7<br>(13.0 , 83.9)	0.07<br>(0.01 , 0.10)	0.0025<br>(0.0007 , 0.0353)	0.0588<br>(0.0010 , 0.1458)	1.04<br>(1.01 , 1.25)	0.06<br>(0.00 , 0.45)	85.41	68.96
**Zhuhai**	Yang Z et al. [3]	2001	65.2<br>(11.2 , 83.5)	0.07<br>(0.01 , 0.10)	0.0086<br>(0.0033 , 0.0916)	0.0602<br>(0.0024 , 0.1457)	1.13<br>(1.08 , 1.33)	0.04<br>(0.00 , 0.23)	171.97	340.67
# **GZ Huangpu**	Zheng X et al. [4]	2008	48.6<br>(13.6 , 83.3)	0.06<br>(0.00 , 0.10)	0.0078<br>(0.0015 , 0.0353)	0.0284<br>(0.0055 , 0.1369)	1.17<br>(1.09 , 2.00)	0.06<br>(0.00 , 0.28)	143.07	280.55
**Guangzhou**	Cao Y et al. [5]	2011-13								
		2011	68.0<br>(15.5 , 83.3)	0.04<br>(0.00 , 0.10)	0.0085<br>(0.0040 , 0.0237)	0.0356<br>(0.0039 , 0.1442)	1.17<br>(1.12 , 1.31)	0.03<br>(0.00 , 0.14)	605.08	1198.32
		2012	61.7<br>(11.9 , 83.1)	0.07<br>(0.01 , 0.10)	0.0174<br>(0.0075 , 0.0448)	0.0415<br>(0.0054 , 0.1407)	1.22<br>(1.15 , 1.42)	0.04<br>(0.00 , 0.20)	517.77	1035.76
		2013	66.8<br>(15.7 , 83.7)	0.07<br>(0.01 , 0.10)	0.0099<br>(0.0039 , 0.0236)	0.0329<br>(0.0048 , 0.1441)	1.14<br>(1.09 , 1.26)	0.03<br>(0.00 , 0.15)	391.61	778.47
**Guangzhou**	Li S et al. [6]	2014	63.8<br>(10.4 , 83.5)	0.07<br>(0.01 , 0.10)	0.0034<br>(0.0014 , 0.1353)	0.0477<br>(0.0009 , 0.1458)	1.06<br>(1.03 , 1.20)	0.03<br>(0.00 , 0.26)	579.46	1054.96
**Guangzhou**	Jing Q et al. [7]	2015	73.2<br>(14.0 , 83.9)	0.07<br>(0.01 , 0.10)	0.0073<br>(0.0027 , 0.0455)	0.0549<br>(0.0031 , 0.1455)	1.10<br>(1.06 , 1.45)	0.05<br>(0.00 , 0.37)	206.43	399.75
**Guangxi Province**										
**Beihai**	Tian X et al. [8]	1980	30.0<br>(12.0 , 80.8)	0.03<br>(0.00 , 0.10)	0.0199<br>(0.0095 , 0.0585)	0.0836<br>(0.0128 , 0.1450)	1.81<br>(1.40 , 3.19)	0.07<br>(0.00 , 0.27)	208.89	418.60
$ **QZ/FCG/HP**	Zhou K et al. [9]	2010-12	78.4<br>(14.5 , 84.2)	0.07<br>(0.02 , 0.10)	0.0019<br>(0.0007 , 0.0096)	0.0594<br>(0.0008 , 0.1450)	1.03<br>(1.02 , 1.09)	0.02<br>(0.00 , 0.14)	184.32	309.73
**Zhejiang Province**										
**Yiwu**	Sun J et al. [10]	2009	45.3<br>(11.1 , 83.3)	0.08<br>(0.02 , 0.10)	0.0413<br>(0.0184 , 0.1223)	0.0415<br>(0.0103 , 0.1333)	1.42<br>(1.27 , 1.76)	0.05<br>(0.00 , 0.19)	217.04	432.83
**Cixi**	Cen D et al. [11]	2004	64.5<br>(11.1 , 83.2)	0.07<br>(0.00 , 0.10)	0.0086<br>(0.0020 , 0.0952)	0.0294<br>(0.0040 , 0.1363)	1.13<br>(1.06 , 1.45)	0.10<br>(0.00 , 0.34)	128.1	252.85
**Hunan Province**										
**Chenzhou**	Gao L et.al. [12]	2005	51.7<br>(10.3 , 83.7)	0.08<br>(0.02 , 0.10)	0.0047<br>(0.0011 , 0.0537)	0.0061<br>(0.0006 , 0.1033)	1.04<br>(1.02 , 1.25)	0.06<br>(0.00 , 0.41)	50.89	88.01
**Guizhou Province**										
**Guiyang**	Gao R et al. [13]	2004-05	63.1<br>(12.8 , 83.6)	0.08<br>(0.02 , 0.10)	0.0105<br>(0.0042 , 0.0580)	0.0458<br>(0.0030 , 0.1442)	1.13<br>(1.07 , 1.52)	0.09<br>(0.00 , 0.44)	669.78	1338.69
**Guiyang**	Tian H et al. [14]	2005	67.1<br>(13.5 , 83.5)	0.08<br>(0.02 , 0.10)	0.0099<br>(0.0038 , 0.0698)	0.0259<br>(0.0011 , 0.1442)	1.11<br>(1.06 , 1.46)	0.10<br>(0.00 , 0.44)	201.76	392.70
& **GY/CJ/LD**	Jiang W et al. [15]	2011	21.2<br>(9.8 , 82.4)	0.07<br>(0.02 , 0.10)	0.0446<br>(0.0032 , 0.1440)	0.0110<br>(0.0009 , 0.1362)	1.15<br>(1.03 , 1.78)	0.20<br>(0.02 , 0.68)	57.11	103.69
**Hainan Province**										

**Danzhou**	Jin Y et al. [16]	2006	70.9 (12.3 , 83.9)	0.07 (0.01 , 0.10)	0.0068 (0.0009, 0.1010)	0.0472 (0.0014 , 0.1441)	1.08 (1.02 , 1.90)	0.10 (0.00 , 0.70)	46.31	83.41
**Yunnan Province**										
**Mengla**	Lu Y et al. [17]	2014	63.9 (12.0 , 83.5)	0.07 (0.01 , 0.10)	0.0064 (0.0013 , 0.0635)	0.0612 (0.0024 , 0.1439)	1.10 (1.03 , 1.51)	0.10 (0.00 , 0.52)	26.49	52.05
**Hekou**	Pu L et al. [18]	2016	65.5 (12.6 , 83.5)	0.07 (0.01 , 0.10)	0.0071 (0.0019 , 0.0569)	0.0569 (0.0027 , 0.1450)	1.11 (1.04 , 1.55)	0.10 (0.00 , 0.50)	39.47	78.80
**Xishuangbanna**	Li L et al. [19]	2019	58.3 (10.6 , 82.8)	0.08 (0.02 , 0.10)	0.0269 (0.0090 , 0.1402)	0.0292 (0.0047 , 0.1427)	1.28 (1.18 , 1.56)	0.05 (0.00 , 0.24)	1174.84	2337.87
**Taiwan Province**										
% **TP/TY/TN**	Lee YH et al. [20]	2010	69.4 (11.8 , 83.7)	0.08 (0.01 , 0.10)	0.0049 (0.0014 , 0.0549)	0.0246 (0.0032 , 0.1411)	1.08 (1.04 , 1.37)	0.10 (0.01 , 0.41)	184.58	362.70
**Kaohsiung**	Tsai JJ et al. [21]	2015-17								
		2015.8-11	68.3 (21.8 , 81.6)	0.06 (0.00 , 0.10)	0.0079 (0.0022 , 0.0455)	0.0774 (0.0145 , 0.1455)	1.20 (1.10 , 1.69)	0.10 (0.01 , 0.43)	127.08	256.07
		2016.2-5	66.1 (11.0, 83.0)	0.07 (0.01 , 0.10)	0.0155 (0.0046 , 0.1169)	0.0397 (0.0056 , 0.1368)	1.21 (1.12 , 1.55)	0.07 (0.00 , 0.31)	111.01	213.90
		2016.9-2017.1	53.3 (13.4 , 83.1)	0.06 (0.01 , 0.10)	0.0141 (0.0036 , 0.0915)	0.0691 (0.0060 , 0.1447)	1.36 (1.10 , 2.17)	0.10 (0.00 , 0.54)	78.94	158.29
		2017.8-9	68.6 (15.7 , 81.3)	0.06 (0.01 , 0.10)	0.0148 (0.0044 , 0.0957)	0.0913 (0.0149 , 0.1465)	1.30 (1.16 , 1.89)	0.10 (0.00 , 0.37)	67.55	131.94
**Kaohsiung/Tainan**	Pan YH et al. [22]	2016								
**Kaohsiung**			66.1 (12.3 , 81.6)	0.07 (0.00 , 0.10)	0.0276 (0.0043 , 0.1341)	0.0684 (0.0159 , 0.1414)	1.40 (1.21 , 2.28)	0.10 (0.00 , 0.42)	961.70	1918.96
**Tainan**			65.5 (12.5 , 83.5)	0.07 (0.00 , 0.10)	0.0181 (0.0022 , 0.1406)	0.0388 (0.0061 , 0.1375)	1.25 (1.09 , 1.99)	0.20 (0.01 , 0.53)	859.07	1712.92
**Hong Kong**	Lee P et al. [23]	2013-15								
		2013	65.4 (49.5 , 80.3)	0.08 (0.01 , 0.10)	0.0037 (0.0012 , 0.0105)	0.0357 (0.0069 , 0.1322)	1.09 (1.06 , 1.20)	0.03 (0.00 , 0.14)	128.19	259.91
		2014	65.5 (63.1 , 67.9)	0.08 (0.01 , 0.10)	0.0028 (0.0012 , 0.0052)	0.0402 (0.0152 , 0.0930)	1.08 (1.06 , 1.15)	0.01 (0.00 , 0.04)	110.84	224.07
		2015	65.5 (63.1 , 67.9)	0.08 (0.01 , 0.10)	0.0026 (0.0009 , 0.0053)	0.0480 (0.0169 , 0.1151)	1.09 (1.06 , 1.17)	0.01 (0.00 , 0.50)	108..42	218.93

Note: # GZ Huangpu represents Huangpu District in Guangzhou city; $ QZ/FCG/HP stands for Qinzhou City, Fangchenggang City and Hepu County in Guangxi; & GY/CJ/LD represents Yunyan District of Guiyang City, Congjiang County of Southeast Guizhou Province and Luodian County of Guizhou Province; % TP/TY/TN indicates Taipei, Taoyuan and Tainan of Taiwan Province.

Table S5: Comparison of -LnL and DIC among different models (Model A-D) fitted to 31 data sets

Survey region	Author	Survey Year	-LnL				DIC				Applicable model
**Guangdong Province**			Model A	Model B	Model C	Model D	Model A	Model B	Model C	Model D	
**Guangzhou**	Huang Y et al. [1]	1981	123.54	121.66	122.80	121.71	249.46	245.36	240.45	245.20	Model C
**Zhuhai**	Li Z et al. [2]	1998	36.20	35.14	35.26	85.41	71.95	69.96	61.68	68.96	Model C
**Zhuhai**	Yang Z et al. [3]	2001	172.46	171.83	172.53	171.97	345.07	343.78	294.39	340.67	Model C
**# GZ Huangpu**	Zheng X et al. [4]	2008	140.82	143.90	140.92	143.07	282.69	287.56	177.13	280.55	Model C
**Guangzhou**	Cao Y et al. [5]	2011-13									
		2011	603.41	605.33	603.45	605.08	1208.26	1210.97	1207.90	1198.32	Model D
		2012	519.23	518.33	518.73	1231.66	1040.36	1038.56	955.25	1035.76	Model C
		2013	392.85	391.98	392.87	391.61	787.48	785.54	717.46	778.47	Model C
**Guangzhou**	Li S et al. [6]	2014	582.55	579.54	582.51	579.46	1164.52	1156.73	966.85	1054.96	Model C
**Guangzhou**	Jing Q et al. [7]	2015	207.67	205.89	207.78	206.43	415.95	411.70	355.98	399.75	Model C
**Guangxi Province**											
**Beihai**	Tian X et al. [8]	1980	208.16	211.03	208.12	208.89	418.28	422.83	417.75	418.60	Model A
**$ QZ/FCG/HP**	Zhou K et al. [9]	2010-12	185.91	184.17	185.87	184.32	372.55	368.74	193.52	309.73	Model C
**Zhejiang Province**											
**Yiwu**	Sun J et al. [10]	2009	221.27	217.26	215.09	217.04	444.86	436.63	433.33	432.83	Model C
**Cixi**	Cen D et al. [11]	2004	127.22	128.71	127.08	128.1	256.27	258.88	218.66	252.85	Model C
**Hunan Province**											
**Chenzhou**	Gao L et.al. [12]	2005	51.99	50.45	50.64	51.48	105.27	101.58	101.25	88.01	Model D
**Guizhou Province**											
**Guiyang**	Gao R et al. [13]	2004-05	672.49	669.62	672.33	669.78	1346.25	1339.97	1339.04	1338.69	Model B
**Guiyang**	Tian H et al. [14]	2005	204.78	201.53	204.61	201.76	410.90	403.67	362.19	392.70	Model C
**& GY/CJ/LD**	Jiang W et al. [15]	2011	59.94	58.17	57.27	57.11	120.93	117.36	80.41	103.69	Model C
**Hainan Province**											
**Danzhou**	Jin Y et al. [16]	2006	45.15	45.10	46.05	46.31	89.21	88.84	53.5	83.41	Model C
**Yunnan Province**											
**Mengla**	Lu Y et al. [17]	2014	26.48	26.05	26.54	26.49	52.97	51.99	46.74	52.05	Model C
**Hekou**	Pu L et al. [18]	2016	39.51	39.24	39.58	39.47	79.88	79.23	72.62	78.80	Model C
**Xishuangbanna**	Li L et al. [19]	2019	1178.3	1174.73	1178.12	1174.84	2358.14	2349.94	2321.00	2337.87	Model C

**Taiwan Province**											
[%] **TP/TY/TN**	Lee YH et al. [20]	2010	184.93	185.17	184.95	184.58	374.11	371.79	278.49	362.70	Model C
**Kaohsiung**	Tsai JJ et al. [21]	2015-17									
		2015.8-11	128.97	130.86	127.49	127.08	260.08	263.05	238.28	256.07	Model C
		2016.2-5	111.18	111.74	109.39	111.01	223.49	225.02	220.42	213.90	Model D
		2016.9-2017.1	78.47	78.95	78.63	78.94	157.37	158.18	155.99	158.29	Model A
		2017.8-9	69.62	71.24	69.28	67.55	141.19	143.92	114.74	131.94	Model C
**Kaohsiung/Tainan**	Pan YH et al. [22]	2016									
**Kaohsiung**			961.22	963.13	961.12	961.7	1924.29	1927.82	1922.57	1918.96	Model C
**Tainan**			858.61	859.77	858.53	859.07	1719.13	1721.15	1718.03	1712.92	Model C
**Hong Kong**	Lee P et al. [23]	2013-15									
		2013	132.91	133.24	132.62	128.19	267.96	268.73	233.79	259.91	Model C
		2014	121.16	120.86	113.46	110.84	244.59	244.05	213.28	224.07	Model C
		2015	118.30	118.23	109.87	108..42	238.80	238.72	213.16	218.93	Model C

Note: [#] GZ Huangpu represents Huangpu District in Guangzhou city; [$] QZ/FCG/HP stands for Qinzhou City, Fangchenggang City and Hepu County in Guangxi; [&] GY/CJ/LD represents Yunyan District of Guiyang City, Congjiang County of Southeast Guizhou Province and Luodian County of Guizhou Province; [%] TP/TY/TN indicates Taipei, Taoyuan and Tainan of Taiwan Province.

3. References

1. Huang Y, Liang J, Lin Z. [Survey of dengue anti-antibody level in population after dengue fever epidemic in Guangzhou]. Guangzhou Medical Journal. 1983(02):20-25.
2. Li Z, Zhang J, Ma H. [Surveillance of Dengue Fever Antibody Levels in People in Zhuhai Port Area]. Chinese Journal of Frontier Health and Quarantine. 1999(03):141-143. DOI: 10.16408/j.1004-9770.1999.03.003.
3. Yang Z, Zhu H, Lin G, Zeng W, Tu C, Ye L, Zhao j, Yao R. [Analysis of dengue antibody levels and related factors in different populations]. Modern Preventive Medicine. 2002(05):694-695. DOI: 10.3969/j.issn.1003-8507.2002.05.066.
4. Zheng X, Wu Y, Zhang M, He A, Li Z, Qu Z, Zhan X. [Sero-epidemiological Investigation of Dengue Fever in Guangzhou]. Journal of Tropical Medicine. 2009,9(12):1397-1399+1404.
5. Cao Y, Jiang L, Xu Y, Jing Q, Cao Q, Di B, Yang Z. [Monitoring and analysis of dengue fever serum antibody levels in Guangzhou from 2011 to 2013]. South China Journal of Preventive Medicine. 2015,41(04):364-366. DOI：10.13217/j.scjpm.2015.0364.
6. Li S, Liao Q, Liang Y, Chen J, You R, Xiong H, Huang K, Rong X. [Study on the risk of dengue virus transmission by blood transfusion in Guangzhou area]. Guangdong Medical Journal. 2017,38(07):1064-1067. DOI: 10.3969/j.issn.1001-9448.2017.07.025.
7. Jing Q, Li Y, Liu J, Jiang L, Chen Z, Su W, Birkhead GS, Lu J, Yang Z. Dengue Underestimation in Guangzhou, China: Evidence of Seroprevalence in Communities With No Reported Cases Before a Large Outbreak in 2014. Open Forum Infect Dis. 2019 Jun 3;6(7):ofz256. doi: 10.1093/ofid/ofz256.
8. Tian X, Ai C, Li C, Wen Y, Liu Y, Xia H. [Epidemiological surveillance of dengue fever in Beihai City, Guangxi]. Journal of Military Medical Sciences. 1985(04):387-392.
9. Zhou K, Chen M, Tan Y, Mo Y, Bi Y. [Serological surveillance of healthy population in Guangxi dengue surveillance sites]. Journal of Applied Preventive Medicine. 2013,19(04):236-237. DOI: 10.3969/j.issn.1673-758X.2013.04.018.
10. Sun J, Luo S, Lin J, Chen J, Hou J, Fu T, Lv H, Chen Z, Cong L, Ling F, et al. Inapparent infection during an outbreak of dengue fever in Southeastern China. Viral Immunol. 2012 Dec;25(6):456-60. doi: 10.1089/vim.2012.0039.
11. Cen D, Wu J, Xu Y. [Application of Serum Bank in the Investigation and Analysis of Dengue Fever Antibody Level]. Chinese Preventive Medicine. 2007(06):734-735. DOI: 10.3969/j.issn.1009-6639.2007.06.022.
12. Gao L, Xiao J, Zhang H, Duan L, Liu F, Dai D, Li J, Deng Z. [Sero-epidemiology investigation of dengue fever in Chenzhou]. South China Journal of Preventive Medicine. 2007(05):34-35. DOI: 10.3969/j.issn.1671-5039.2007.05.011.
13. Gao R, Zhou X, Zhou C, Han Y, Luo J, Qin J. [Serological Epidemiological Research on Dengue Virus Antibodies among Personnel at Guiyang Port]. Chinese Journal of Frontier Health and Quarantine. 2006(S1):57-59. DOI: 10.3969/j.issn.1004-9770.2006.z1.017.
14. Tian H, Han Y, Dai A, Fu D, Zhou Y, Zhou N, Gao R. [The Investigation of the Population Infected with Dengue Virus at Guiyang

Port and other Close Areas]. Journal of Travel Medicine Science. 2007(04):28-29. DOI: 10.3969/j.issn.1006-7159.2007.04.009.

15. Jiang W, Zhou J, Tang G, Zhuang Y, Yun C, Fu L. [Investigation on Dengue Fever Infection among Healthy Population in Some Counties and Cities in Guizhou Province]. Guizhou Medical Journal. 2013,37(02):164-165. DOI: 10.3969/j.ISSN.1000-744X.2013.02.028.

16. Jin Y, Sun L, Zeng X, Wu W, Ma Y, Su X, Lao S, Chen Y, Li Z. [Sero-epidemiological survey and analysis on dengue fever in Hainan Province]. China Tropical Medicine. 2007(11):2007-2008. DOI: 10.3969/j.issn.1009-9727.2007.11.014.

17. Lu Y, Liu Y, Ji R, Luo Z, Zhang Q, Zhou L, Yang X. [Serological surveillance on arboviral diseases among exit-entry population at Sino-Laos port]. Chinese Journal of Frontier Health and Quarantine. 2016,39(03):180-182. DOI: 10.16408/j.1004-9770.2016.03.008.

18. Pu L, Chen J, Zhang Q, Zhang C, Li Z, Liu W, Wu Z. [Aedes surveillance and dengue fever serological survey at Sino-Vietnam Hekou-Laocai ports]. Chinese Journal of Frontier Health and Quarantine. 2018,41(04):255-257+268. DOI: 10.16408/j.1004-9770.2018.04.008.

19. Li L, Li Y, Lu S, Dong J, Xu H, Zhang Q, Weng R, Yin Y, He R, Fang P, et al. Epidemiological survey and screening strategy for dengue virus in blood donors from Yunnan Province. BMC Infect Dis. 2021 Jan 22;21(1):104. doi: 10.1186/s12879-021-05810-8.

20. Lee YH, Hsieh YC, Chen CJ, Lin TY, Huang YC. Retrospective Seroepidemiology study of dengue virus infection in Taiwan. BMC Infect Dis. 2021 Jan 21;21(1):96. doi: 10.1186/s12879-021-05809-1.

21. Tsai JJ, Liu CK, Tsai WY, Liu LT, Tyson J, Tsai CY, Lin PC, Wang WK. Seroprevalence of dengue virus in two districts of Kaohsiung City after the largest dengue outbreak in Taiwan since World War II. PLoS Negl Trop Dis. 2018 Oct 24;12(10):e0006879. doi: 10.1371/journal.pntd.0006879.

22. Pan YH, Liao MY, Chien YW, Ho TS, Ko HY, Yang CR, Chang SF, Yu CY, Lin SY, Shih PW, Shu PY, Chao DY, Pan CY, Chen HM, Perng GC, Ku CC, King CC. Use of seroprevalence to guide dengue vaccination plans for older adults in a dengue non-endemic country. PLoS Negl Trop Dis. 2021 Apr 1;15(4):e0009312. doi: 10.1371/journal.pntd.0009312.

23. Lee P, Yeung ACM, Chen Z, Chan MCW, Sze KH, Chan PKS. Age-specific seroprevalence of dengue infection in Hong Kong. J Med Virol. 2018 Sep;90(9):1427-1430. doi: 10.1002/jmv.25216.
